# Diverse *w*Mel variants of *Wolbachia pipientis* differentially rescue fertility and cytological defects of the *bag of marbles* partial loss of function mutation in *Drosophila melanogaster*

**DOI:** 10.1093/g3journal/jkab312

**Published:** 2021-09-07

**Authors:** Jaclyn E Bubnell, Paula Fernandez-Begne, Cynthia K S Ulbing, Charles F Aquadro

**Affiliations:** Department of Molecular Biology and Genetics, Cornell University, Ithaca, NY 14850, USA

**Keywords:** *Wolbachia*, germline stem cell, bam, oogenesis, differentiation

## Abstract

In *Drosophila melanogaster*, the maternally inherited endosymbiont *Wolbachia pipientis* interacts with germline stem cell genes during oogenesis. One such gene, *bag of marbles* (*bam*) is the key switch for differentiation and also shows signals of adaptive evolution for protein diversification. These observations have led us to hypothesize that *W. pipientis* could be driving the adaptive evolution of *bam* for control of oogenesis. To test this hypothesis, we must understand the specificity of the genetic interaction between *bam* and *W. pipientis*. Previously, we documented that the *W. pipientis* variant, *w*Mel, rescued the fertility of the *bam^BW^* hypomorphic mutant as a transheterozygote over a *bam* null. However, *bam^BW^* was generated more than 20 years ago in an uncontrolled genetic background and maintained over a balancer chromosome. Consequently, the chromosome carrying *bam^BW^* accumulated mutations that have prevented controlled experiments to further assess the interaction. Here, we used CRISPR/Cas9 to engineer the same single amino acid *bam* hypomorphic mutation (*bam^L255F^*) and a new *bam* null disruption mutation into the *w^1118^* isogenic background. We assess the fertility of wildtype *bam*, *bam^L255F^/bam^null^* hypomorphic, and *bam^L255F^/bam^L255F^* mutant females, each infected individually with 10 *W. pipientis w*Mel variants representing three phylogenetic clades. Overall, we find that all of the *W. pipientis* variants tested here rescue *bam* hypomorphic fertility defects with *w*MelCS-like variants exhibiting the strongest rescue effects. In addition, these variants did not increase wildtype *bam* female fertility. Therefore, both *bam* and *W. pipientis* interact in genotype-specific ways to modulate female fertility, a critical fitness phenotype.

## Introduction

*Wolbachia pipientis* is a maternally inherited endosymbiotic bacteria that infects over 65% of insect species and manipulates reproduction in a myriad of ways in order to ensure its transmission through the female germline (Werren *et al.* 2008; Lindsey 2020; Ote and Yamamoto 2020). The phenotypes *W. pipientis* induces in its hosts include cytoplasmic incompatibility (CI), in which embryos of matings between infected males and uninfected females die; male killing, in which male embryos die; feminization of embryos; and manipulation of germline stem cell (GSC) differentiation in order to increase female fertility (Werren *et al.* 2008). While *W. pipientis* manipulates its host to increase its own transmission, some *W. pipientis* also provide the host protection against viruses, increase fecundity, modulate thermal preference, and increase longevity (Dedeine *et al.* 2001; Chrostek *et al.* 2013; Arnold *et al.* 2019; Truitt *et al.* 2019; Hague *et al.* 2020; López-Madrigal and Duarte 2020).

Understanding the genetic mechanisms that *W. pipientis* uses to manipulate its host has been of immense interest to both basic and applied fields of study. *W. pipientis* is of particular interest as a control for disease vectors such as mosquitoes due to its ability to sweep through a population (due to CI) and then protect the insect from viruses that can also cause human illness such as Dengue, Zika, and Chikungunya (Moreira *et al.* 2009; Hoffmann *et al.* 2011; Dutra *et al.* 2016; Utarini *et al.* 2021). However, it has been difficult to perform genetic studies of *W. pipientis* function to understand the mechanisms of these host-microbe interactions since *W. pipientis* is an obligate endosymbiont and cannot be cultured. Over the past few years, multiple groups have utilized bioinformatic and in vitro screens to identify candidate *W. pipientis* loci that modulate *Drosophila* phenotypes (Ote *et al.* 2016; Le Page *et al.* 2017). These loci have then been expressed in *Drosophila* as transgenes. Through these methods, the *W. pipientis* genes *cifA* and *cifB* (orthologs in *w*Mel and *w*Pip) that cause CI have been identified and functionally validated in *D. melanogaster* (Beckmann *et al.* 2017; Le Page *et al.* 2017). These transgenic tools have been used to further define the CI phenotype (Shropshire *et al.* 2018), investigate the consequences of genetic variation at *cifA* and *cifB* on CI (Shropshire *et al.* 2021), and identify other host phenotypes affected by *cifA* and *cifB* (Deehan *et al.* 2021). Another group has identified the *W. pipientis* TomO locus which is one of likely multiple *W. pipientis* loci that interact with *Sex lethal*, a GSC gene that controls GSC maintenance and sex determination (Ote *et al.* 2016).

Previously, we reported that the *W. pipientis* variant *w*Mel genetically interacts with another *D. melanogaster* GSC gene, *bag of marbles* (*bam*). Infection with the *w*Mel variant rescues the fertility defect of females transheterozygous for the classic *bam* hypomorphic allele (*bam^BW^*) and a classic *bam* null allele (*bam^Δ59^*) (Flores *et al.* 2015). *Bam* is the switch for GSC daughter differentiation in *D. melanogaster* females and the switch for terminal spermatocyte differentiation in males (McKearin and Spradling 1990; Insco *et al.* 2012; Ting 2013). Although *bam* function is essential for gametogenesis, and *bam* is both necessary and sufficient for GSC daughter differentiation, we observe that it is evolving under positive selection for amino acid divergence in an episodic manner across the *Drosophila* genus (Bauer DuMont *et al.* 2007; Choi and Aquadro 2014). We see particularly strong bursts of amino acid changes at *bam* in the *D. melanogaster* and *D. simulans* lineages (Bauer DuMont *et al.* 2007). This episodic pattern of positive selection is consistent with selective pressures that are present in some lineages and not others and that may come and go over time. This is similar to the nature of infections in natural populations, where *W. pipientis* variants are known to infect populations, but also can be lost and replaced fairly often and on both long and short time scales (Bailly-Bechet *et al.* 2017).

Our observation that *W. pipientis* and *bam* genetically interact led us to hypothesize that *W. pipientis* may drive the adaptive evolution of *bam* (Flores *et al.* 2015). Since *W. pipientis* is a known manipulator of reproduction, *W. pipientis* may be in conflict with *bam* and other GSC genes for control of oogenesis. While it may seem favorable to host fitness if *W. pipientis* manipulates GSC regulation to increase reproduction, and thus not present an evolutionary conflict, it may not be beneficial for the host if oogenesis is regulated by *W. pipientis*. For example, *W. pipientis* may promote oogenesis when it is not favorable for the host to reproduce due to environmental or physiological factors. To further understand if *W. pipientis* could be in genetic conflict with *bam* and drive its adaptation, we believe it is necessary to better understand the genetic interaction between *bam* and *W. pipientis*.

Fully sequenced variants of *W. pipientis* that infect *D. melanogaster* have been found to cluster phenotypically and phylogenetically into two distinct groups of clades: *w*Mel-like variants (including *w*Mel and *w*Mel2) and *w*MelCS-like variants (including *w*MelCS, *w*MelCS2, and *w*MelPop) that are estimated to have diverged 80,000 fly generations before present (Richardson *et al.* 2012; Chrostek *et al.* 2013). The *w*MelCS-like variants predominated in *D. melanogaster* originally but have largely (although not completely) been replaced by *w*Mel-like variants world-wide in the late 20th century (Riegler *et al.* 2005). The *w*MelCS-like variants provide stronger viral protection, reach higher intracellular bacterial titers in males, and often shorten lifespan compared to the more benign *w*Mel-like variants that now predominate. In addition, uninfected *D. melanogaster* and those infected with *w*Mel-like variants have a higher temperature preference than *D. melanogaster* infected with *w*MelCS-like variants (including *w*MelPop) (Truitt *et al.* 2019).

To assess the specificity of our initial observation that *w*Mel rescues the female fertility phenotype of the *bam^BW^*/*bam^Δ59^* hypomorphic mutant, we made use of a genetic tool for functionally assessing *W. pipientis* variation generated by Chrostek *et al.* (2013), a set of *w^1118^* isogenic lines individually infected with 10 diverse *w*Mel *W. pipientis* variants from two of the *w*Mel-like clades and the *w*MelCS-like clade defined by Richardson *et al.* (2012). The 10 *W. pipentis* variants that we analyze thus include both phenotypic classes of *w*Mel variants: the *w*MelCS-like variants which are higher titer, reduce host lifespan, and confer higher viral resistance and lower thermal preference in contrast to the more recent *w*Mel-like variants.

Since fertility is a phenotype affected by genome-wide variation and both the *bam^BW^* and *bam^Δ59^* alleles were generated in nonisogenic backgrounds, we sought to control the *Drosophila* genetic background to better isolate the effect of *W. pipientis* variation and *bam* genotype on fertility. We used CRISPR/Cas9 to edit the single amino acid change of the original *bam^BW^* hypomorph (Ohlstein *et al.* 2000) into the *w^1118^* isogenic background, and also to create a new *bam* null allele in the same genetic background. In addition, we had previously not been able to determine the phenotype of *bam^BW^*/*bam^BW^* females, as the *bam^BW^* allele was isolated over 20 years ago and then maintained over a balancer chromosome which allowed the mutation carrying chromosome to accumulated recessive lethal mutations. Thus, we also used these new lines to analyze *bam* hypomorph and null alleles as homozygotes and transheterozygotes.

We confirmed that the amino acid replacement results in the *bam* hypomorphic phenotype previously described when expressed over a *bam* null allele in uninfected females (Flores *et al.* 2015). Interestingly, we further show that uninfected females homozygous for the *bam* hypomorphic allele do not exhibit GSC tumors or show reduced fertility compared to wild-type females. We then assessed the effect of infection by the individual *W. pipientis* variants by crossing the cytoplasmic maternal backgrounds infected with each *W. pipientis* variant into our *w^1118^ bam* hypomorph line thereby maintaining the same nuclear background (except for the *bam* locus).

We find that all *w*Mel *W. pipientis* variants tested here do not increase fertility in the wildtype *bam* background, but all *w*Mel *W. pipientis* variants rescue the fertility of *bam* mutant females, with *w*MelCS-like *W. pipientis* showing the highest rescue effects. Therefore, the fertility rescue of the *bam* hypomorph by *W. pipientis* is not due to an overall increase in fertility modulated by *W. pipientis*, but a specific interaction between *bam* and *W. pipientis* genotypes.

## Materials and methods

### Fly stocks and rearing

Prior to experiments, we raised fly stocks on standard cornmeal molasses food at room temperature. We used yeast glucose food for fertility assays. During experiments, we maintained crosses and stocks in an incubator at 25°C with a 12-h light–dark cycle. The lines carrying the classic *bam* alleles, *bam^Δ59^* (null) and *bam^BW^* (hypomorph) are described on Flybase (Thurmond *et al.* 2019) and in Flores *et al.* (2015). The *W. pipientis* infected *w^1118^* isogenic lines used in this study were generous gifts from Luis Texiera and described in Chrostek *et al.* (2013). We used CantonS males for the *w^1118^*; *bam^L255F^*/*bam*^null^ and *w^1118^*; *bam*^+^/*bam*^+^ fertility assays. We used *w^1118^* isogenic males for the *w^1118^*; *bam^L255F^*/*bam^L255F^* fertility assays. We verified that males were uninfected with *W. pipientis* by endpoint PCR and qPCR using primers described below. To generate the *bam* hypomorphic lines infected with the 10 different *W. pipientis* variants, we crossed females from the *w^1118^*; TM2/TM6 stock infected with each variant to *w^1118^*; *bam^L255F^*/TM6 males.

### Generation of bam alleles with CRISPR/Cas9

We engineered the new *bam^L255F^* hypomorph and *bam* null alleles described in this study in the *w^1118^* isogenic background using synthetic gRNAs and Cas9 protein as follows.

#### gRNAs

We used the flyCRISPR target finder to choose gRNAs with zero predicted off-targets in the *D. melanogaster* genome (Supplementary Table S1). We then ordered synthetic gRNAs (sgRNA) from Synthego.

#### Cloning

We generated all PCR products for cloning with NEB High Fidelity Q5 master mix, and gel extracted and purified PCR products using the Qiagen MinElute gel extraction kit. We created the donor plasmid for homology directed repair using the NEB HiFi assembly cloning kit and the pHD-attP-DsRed vector from flyCRISPR (Gratz *et al.* 2014). For plasmid prep, we used Qiagen plasmid plus midi-prep kit and sequenced plasmids with Sanger sequencing (Cornell BRC Genomics Core). We ordered primers for PCR, sequencing, and cloning from IDTDNA (Supplementary Table S2).

#### Donor sequences

To generate the hypomorphic *bam^L255F^* allele we designed a single stranded oligo donor (ssODN) that contained the single amino acid mutation of the original *bam^BW^* hypomorphic allele (CTT->TTT) as well as a single synonymous change to the closest preferred codon in order to kill the gRNA site upon homology directed repair. We used 80 bp of homology on each side of our targeted change (Supplementary Table S1).

To generate the null *bam^null-In2-3xP3-DsRed^* allele, we amplified 1.5 kb homology arms from the *w^1118^* isogenic line and cloned them into the pHD-attP-DsRed vector from flyCRISPR (Supplementary Table S3). As we did not need the attP site in our line, we amplified the 3xP3-DsRed cassette from the plasmid and assembled the homology arms, 3xP3-DsRed, and the original vector backbone with the NEB HiFi assembly kit, thereby removing the attP site.

#### Injections

All CRISPR/Cas9 injections were sourced to Genetivision and were done in the *w^1118^* isogenic line. The injection mix contained plasmid or ssODN donor, sgRNAs, Cas9 protein (Synthego), and an siRNA for Lig4 (IDT DNA, Supplementary Table S1).

For the *bam^null-In2-3xP3-DsRed^* allele, we screened for the eye color cassette in F1’s in house using a Nightsea fluorescent system (DsRed filters). For the *bam^L255F^* allele, the edited nucleotide change disrupts an AflII restriction site, allowing us to screen F1’s by PCR (Promega GoTaq mastermix) followed by restriction digests with AflII (NEB). We prepared genomic DNA using the Qiagen PureGene kit.

We backcrossed females of all CRISPR/Cas9 mutants to *w^1118^* isogenic males for three generations, and then crossed the mutants to the *w^1118^*; TM2/TM6 line to maintain the *bam* mutants over the TM6 balancer. All CRISPR/Cas9 edits in the lines were confirmed by Sanger sequencing (Cornell BRC Genomics Facility).

### PCR assays to detect *W. pipientis*

To test for the presence of *W. pipientis*, we used the Zymo quick DNA miniprep kit to prepare DNA from three replicate samples each with three female flies. For each sample, we used three different primers for endpoint or qPCR (Supplementary Table S1). We used the common *wsp* primers (Flores *et al.* 2015), a primer pair targeting *DprA*, and a highly sensitive primer pair to the ARM repeat (Schneider *et al.* 2014) for endpoint PCR, modified here for qPCR (Supplementary Table S2).

### Immunostaining

We performed immunostaining as described in Aruna *et al.* (2009) and Flores *et al.* (2015). Briefly, we dissected ovaries in ice cold 1X PBS and pipetted the tissue up and down to improve antibody permeability. We fixed ovaries in 4% paraformaldehyde (EMS), washed with PBST (1X PBS, 0.1% Triton-X 100), blocked in PBTA (1X PBS, 0.1% Triton-X 100, 3% BSA) (Alfa Aesar), and then incubated in the appropriate primary antibody overnight. We then washed, blocked, and incubated in the appropriate secondary antibody for 2 h then washed and mounted in ProLong Glass NucBlue for imaging (ThermoFisher). We used an anti-Vasa antibody from Santa Cruz Biotechnology at 1:200 (Cat# sc-30210, RRID: AB_793874, anti-rabbit) and a goat anti-rabbit secondary antibody from Thermo Fisher Scientific at 1:500 (Alexa Fluor Plus 488, Cat# A32731, RRID: AB_2633280). We imaged ovaries on a Zeiss i880 confocal microscope with 405 and 488 nm laser lines at 40X (Plan-Apochromat 1.4 NA, oil) (Cornell BRC Imaging Core). We analyzed and edited images using Fiji (ImageJ).

### Assays for nurse cell positive egg chambers

We used the same assay we previously described to assess the rescue of the original *bam^BW^/bam ^Δ59^* hypomorphic cytological phenotype by *W. pipientis* (Flores *et al.* 2015). We dissected ovaries from mated 2–4 days old *bam^L255F^*/*bam^null-In2-3xP3-DsRed^* females for all *W. pipientis* variants and the uninfected control in PBS. Ovaries were fixed in 4% paraformaldehyde (EMS), washed with PBST (1X PBS, 0.1% Triton-X 100), and then mounted in ProLong Glass with NucBlue (ThermoFisher). We imaged ovaries on a Zeiss i880 confocal microscope with a 405 nm laser line at 10X (C-Apochromat 0.45 NA, water) and 40X (described above). We analyzed and edited images using Fiji (ImageJ). Developing ovaries consist of cysts containing differentiated nurse cells which are polyploid and feature large, easily identifiable nuclei. In contrast, GSC daughter differentiation is blocked in *bam* loss-of-function ovaries resulting in cysts filled with small GSC-like cells with small nuclei. To quantify the rescue of *bam's* differentiation function, we counted the number of nurse cell positive egg chambers (cysts) per ovary for uninfected females and females infected with each *W. pipientis* genotype (as these ovaries are small and underdeveloped, they stay intact during the fixation and washing steps).

### Fertility assays

We used the following *w^1118^* isogenic *bam* genotypes for the fertility assays: *w^1118^*; *bam*^+^/*bam*^+^, *w^1118^*; *bam^L255F^*/*bam^null-In2-3xP3-DsRed^*, and *w^1118^*; *bam^L255F^*/*bam^L255F^*. In addition, we performed a small control fertility assay using combinations of the alleles described above and the classic *bam^BW^* hypomorph and *bam^Δ59^* alleles.

We performed the fertility assays for *w^1118^*; *bam*^+^/*bam*^+^ and *w^1118^*; *bam^L255F^*/*bam^null-In2-3xP3-DsRed^* in three batches to reduce technical error from too large of an experiment. We included separate uninfected controls in each batch of *W. pipientis* variants and used these to make statistical comparisons of mean progeny per female.

We performed all fertility assays (except for the small *bam^BW^* and *bam^Δ59^* analysis) as follows (and described by Flores *et al.* 2015):

We collected virgin females and aged them 2–3 days, only using flies that eclosed within 48 h of each other to reduce developmental variation. We collected virgin males uninfected with *W. pipientis* and aged them for 2–3 days. We distributed males from different bottles across the female genotypes to control for any bottle effects. We individually crossed virgin females to two virgin males. The trio was allowed to mate for 8 days, and then flipped to new vials. After the second 8 days, the trio was cleared. The progeny for each trio was counted every other day to get the total adult progeny per female. Progeny per female is reported in increments by day that reflect the days after the first progeny eclosed. For example, we report total progeny for days 1–9 which are the progeny counted on day 1 of eclosion to day 9 of eclosion.

For the control fertility assay containing the *bam^BW^* hypomorph and *bam^Δ59^* alleles, we collected and aged flies as described above, except the progeny per trio was counted only once at the end of the experiment.

### Egg laying assay

We collected virgin females for each genotype and *W. pipientis* variant and CantonS virgin males and aged them as described above for the fertility assays. We allowed each trio to mate for 24 h on grape juice agar supplemented with yeast before flipping them to a new grape juice agar vial. We counted the eggs laid within 24 h and repeated this for 3 days.

### Statistics

For the nurse cell assay, egg laying assay, and fertility assays we used estimation statistics to assess the mean difference (effect size) of nurse cells, eggs, and adult progeny between infected and uninfected lines. All counts for nurse cells, eggs, and progeny from these assays are reported in Supplementary File S1. All estimation statistics were done using the dabest package in Python (v. 0.3.1) with 5000 bootstrap resamples (Ho *et al.* 2019). Estimation statistics provide a nonparametric alternative to other statistical tests of the difference of the mean (*e.g.*, ANOVA), and allow us to understand the magnitude of the effect of *W. pipientis* variation on the *bam* phenotype. We display the data with a swarm plot that shows all of the data points and either a Cumming estimation plot (for more than two sample comparisons) or a Gardner-Altman plot (two sample comparisons) that shows the effect size for each sample compared to the control and 95% bootstrap confidence interval. In text, we report significance as a mean difference (effect size) outside the 95% bootstrap confidence interval.

## Results

### *bam^L255F^* is a hypomorphic *bam* allele

**Figure 1:**
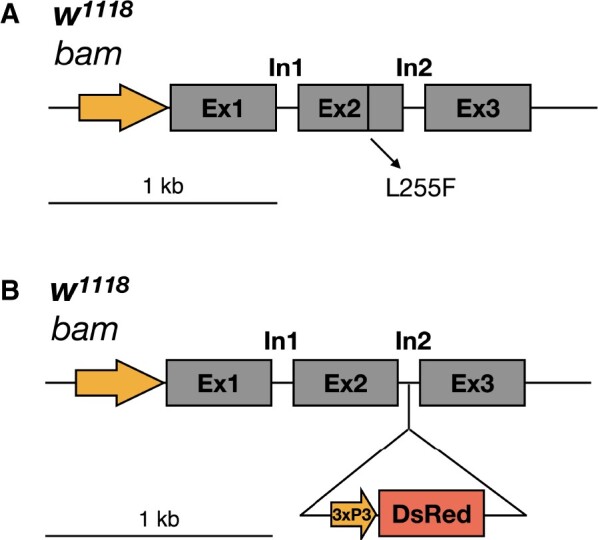
Design for recreating the classic *bam^BW^* hypomorph allele and a new *bam* null allele in the *w^1118^* isogenic background with CRISPR/Cas9. (A) Schematic of the *bam* gene region showing the single gRNA target site in the second exon and the hypomorphic missense mutation. (B) Schematic of the *bam* gene region showing the single gRNA target site in the second intron with the 3xP3-DsRed eye marker to create a *bam* null allele.

We used CRISPR/Cas9 to recreate the same *bam* hypomorphic mutation as the original *bam^BW^* allele (Ohlstein *et al.* 2000) and a new *bam* null allele in the isogenic *w^1118^* background (Figure 1). Therefore, we have two new *bam* mutant alleles, *bam^L255F^* and *bam^null-In2-3xP3-DsRed^* in the same genetic background so we can compare wildtype bam fertility, the transheterozygous hypomorph/null fertility (as we did previously with *bam^BW^/bam^Δ59^*) as well as assess the phenotype of the homozygous *bam* hypomorphic genotype which has never been assessed before.

**Figure 2:**
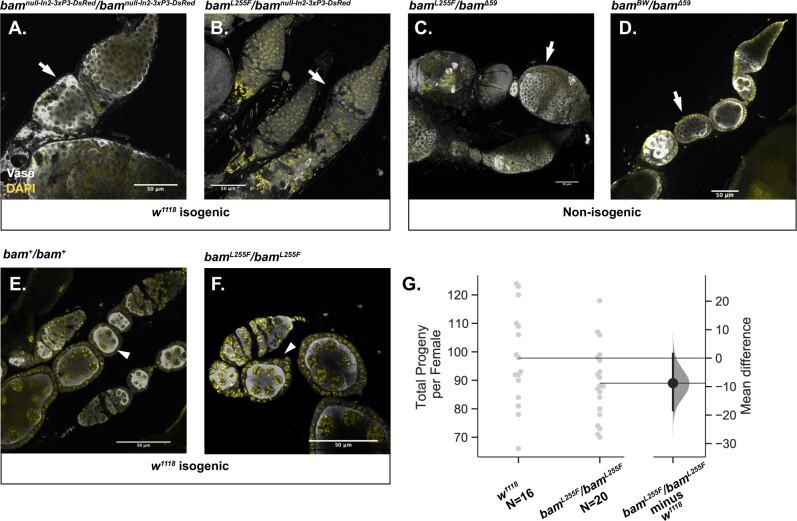
The *bam^BW^* hypomorphic allele and a novel *bam* null allele in the *w^1118^* isogenic background recapitulates the classic *bam* phenotypes. (A) The newly generated *bam^null-In2-3xP3-DsRed^/bam^null-In2-3xP3-DsRed^* genotype results in tumorous ovaries (arrow). (B) The recreated *bam^L255F^* mutation over *bam^null-In2-3xP3-DsRed^* exhibits tumorous ovaries in the *w^1118^* isogenic background (arrow). (C) The recreated *bam^L255F^* mutation over the classic *bam*^Δ59^ null allele exhibits tumorous ovaries (arrow). (D) The classic *bam^BW^/bam^Δ59^* hypomorphic genotype exhibits tumorous ovaries (arrow). (E) Ovaries from the *w^1118^* isogenic line wildtype for *bam* do not exhibit any germline tumors and show developing egg chambers with large nurse cell nuclei (arrowhead). (F) *bam^L255F^*/*bam^L255F^* homozygotes do not exhibit tumorous ovaries, indicating two copies of the partial loss of function *bam^L255F^* mutation is sufficient for GSC daughter differentiation. (G) *w^1118^* females and *bam^L255F^*/*bam^L255F^* females do not show a significant difference in fertility (mean difference of progeny per female), further indicating that two copies of *bam^L255F^* is sufficient for *bam* function.

We evaluated if the *w^1118^*; *bam^L255F^* allele recapitulated the *bam^BW^/bam^Δ59^* mutant phenotypes by assessing the phenotype of a transheterozygous *w^1118^*; *bam^L255F^* hypomorph over a *bam* null allele. Since we wanted all of our alleles in the same genetic background and there were no existing *bam* null alleles in the *w^1118^* isogenic background, we used CRISPR/Cas9 to knock in a 3xP3-DsRed cassette into the second intron of *bam*, which resulted in a *bam* null allele in the same *w^1118^* background marked with a trackable eye marker (*w^1118^*; *bam^null-In2-3xP3-DsRed^*, Figure 1B). Females homozygous for this *bam^null-In2-3xP3-DsRed^* allele exhibit the expected tumorous ovary phenotype with no developing nurse cell-positive cysts and are sterile (Figure 2A, fertility data not shown).

**Figure 3:**
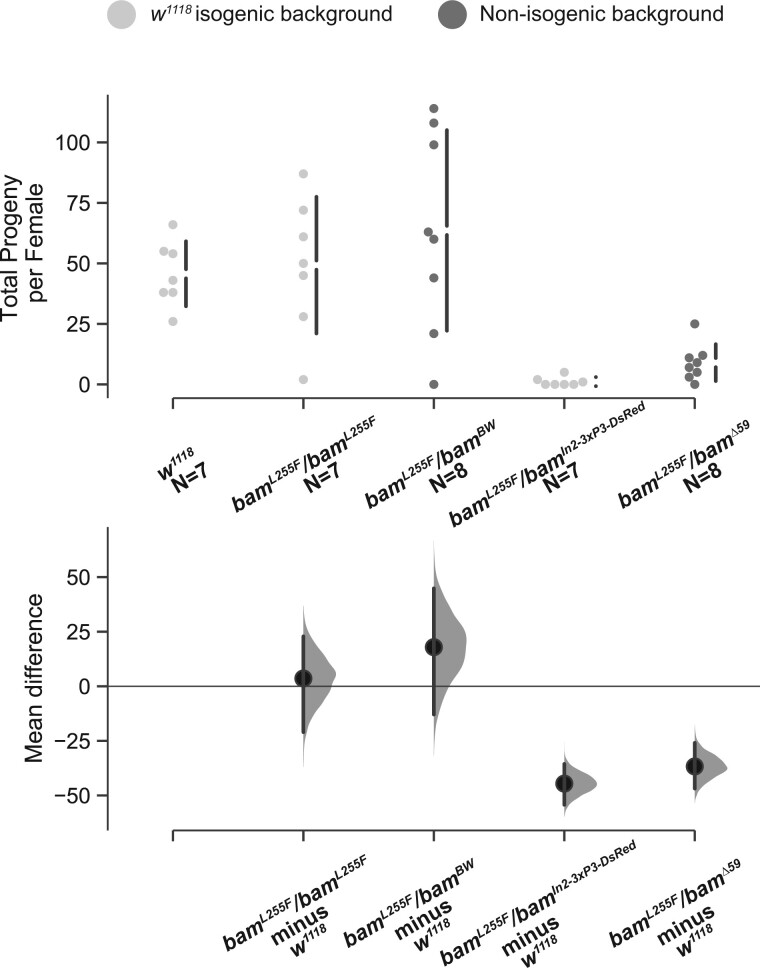
Female fertility of genotypes containing the *w^1118^; bam^L255F^* hypomorphic allele are consistent with those of classic alleles. Swarm and Cumming estimation plots of a control fertility experiment showing that the fertility of *bam^L255F^*/*bam^L255F^* females is not significantly different from *w^1118^* (mean difference = 3.6), nor are *bam^L255F^*/*bam^BW^* females (mean difference = 17.9). *bam^L255F^*/*bam^null-In2-3xP3-DsRed^* females have significantly lower fertility compared to wildtype (mean difference = −44.6, bootstrap 95% confidence interval, effect size), as do *bam^L255F^*/*bam^Δ59^* females (mean difference = −36.7, bootstrap 95% confidence interval, effect size).

We then assessed the phenotype of the *bam^L255F^* allele as a transheterozygous mutant over our new *bam^null-In2-3xP3-DsRed^* in the same *w^1118^* isogenic background. We observed a large fertility defect, with some sterile and some weakly fertile females with only 1–5 total progeny (Figure 3). The *bam^L255F^*/*bam^null-In2-3xP3-DsRed^* ovaries exhibited the expected tumorous ovary phenotype, with the presence of some differentiating, nurse cell positive cysts indicating that *bam* is partially functional, and thus that this single amino acid change fully explains the original *bam^BW^* hypomorphic phenotype (Figure 2B). To further verify the nature of the *bam^L255F^* allele, we assessed its phenotype over one of the classic *bam* null alleles that we previously used to study the interaction between *bam* and *W. pipientis, bam^Δ59^*. The *bam^Δ59^* null allele is a nearly full deletion of the *bam* coding sequence. We examined ovaries from *bam^L255F^*/*bam^Δ59^* females and observed the tumorous ovary phenotype with some developing cysts as has been previously documented for *bam^BW^/bam^Δ59^* and additionally shown here (Figure 2, C and D). We measured the fertility of *bam^L255F^*/*bam^Δ59^* females and observed a large fertility defect, consistent with the fertility of the *bam^L255F^*/*bam^null-In2-3xP3-DsRed^* genotype (Figure 3). Therefore, the *bam^L255F^*/*bam^null-In2-3xP3-DsRed^* cytological and fertility phenotypes are consistent with the previously documented *bam* hypomorph phenotypes.

As the *bam^BW^* allele is homozygous lethal due to accumulated recessive lethal mutations on the third chromosome, we used our *bam^L255F^* allele to ask if the homozygous *bam^L255F^* females also had a hypomorphic phenotype. We asked if *bam^L255F^*/*bam^L255F^* females also exhibited the classic *bag of marbles* cytological phenotype and reduced fertility. In contrast to the tumorous ovary phenotype of *bam^BW^/bam^Δ59^* and *bam^L255F^*/*bam^null-In2-3xP3-DsRed^*, a result of a defect in *bam’*s differentiation function which causes GSC-like cells to over-proliferate, we found no evidence of tumorous cysts in ovaries of *bam^L255F^*/*bam^L255F^* females (Figure 2F). These ovaries resembled those of a wildtype *bam* background, with all developing egg chambers featuring nurse cells (Figure 2E). Previously we were unable to compare the fertility of the *bam^BW^* hypomorph to wildtype *bam* fertility as fertility is greatly affected by different genetic backgrounds. We compared the fertility of the *w^1118^*; *bam^L255F^*/*bam^L255F^* females to *w^1118^*; *bam*^+^/*bam*^+^ females and found that the mean difference in total progeny per female between the two genotypes was not significantly different (Figure 2G). While we did not measure all aspects of *bam* function of this genotype, the fertility and cytological data here indicate that the *bam^L255F^*/*bam^L255F^* genotype does not cause a severe *bam* mutant phenotype, as we observed previously for the transheterozygous *bam^BW^* hypomorph (*bam^BW^/bam^Δ59^*). Therefore, two copies of the *bam^L255F^* allele are sufficient for GSC daughter differentiation and fertility.

Although we cannot make strong claims about the effect of *bam* genotype on fertility between lines with different genetic backgrounds, we did perform a small control experiment to ask if our new *bam^L255F^* hypomorphic allele over the classic *bam^BW^* hypomorphic allele also show similar fertility to wildtype females as we observed for *bam^L255F^*/*bam^L255F^* females*.* We observed that *bam^L255F^*/*bam^BW^* females and homozygous *bam^L255F^* females did not show significantly different fertility from *w^1118^ bam^+^/bam^+^* females (Figure 3). Further, we asked if our new *bam^L255F^*/*bam^null-In2-3xP3-DsRed^* genotype showed a similar fertility defect to *bam^L255F^*/*bam^Δ59^* (classic null) and found that both showed severely reduced fertility compared to wildtype (Figure 3). These data further confirm that our new *bam^L255F^* and *bam^null-In2-3xP3-DsRed^* alleles behave similarly to alleles we have used in the past to study the interaction between *bam* and *W. pipientis.*

Since we were able to successfully recapitulate the partial-loss of function *bam* cytological and fertility phenotypes with the *bam^L255F^*/*bam^null-In2-3xP3-DsRed^* genotype as we have previously described (Flores *et al.* 2015), but we did not observe the *bam* partial loss of function ovarian cytological phenotype in the *bam^L255F^*/*bam^L255F^* females, we focused primarily on the effect of *W. pipientis* variation on the *bam^L255F^*/*bam^null-In2-3xP3-DsRed^* female fertility and cytological phenotypes.

For convenience and readability, we will frequently refer to the *w^1118^*; *bam^null-In2-3xP3-DsRed^* allele as *bam^null^* and the *w^1118^*; *bam^L255F^* allele as *bam^L255F^.*

### Infection with the 10 different *w*Mel variants we assayed does not increase *w^1118^*; *bam^+^/bam^+^* isofemale fertility

**Figure 4:**
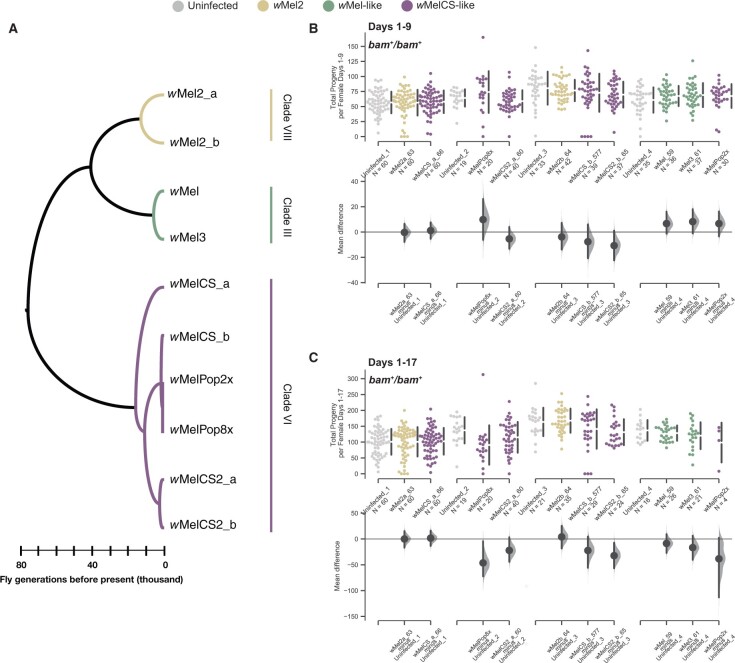
Total progeny per female and mean difference of progeny per female for the *w^1118^*; *bam*^+^/*bam*^+^ genotype infected with 10 *W. pipientis w*Mel variants compared to uninfected. (A) Cladogram adapted from Chrostek *et al.* (2013) showing the relationships and clade assignments for the *W. pipientis* variants tested in this study. (B) Swarm and Cumming estimation plots showing the total progeny per female for each *W. pipientis* infected line counted on days 1–9 post eclosion. As the fertility assays were performed in batches, each batch is compared to its own uninfected control. No *W. pipientis* variant was associated with a significant difference in fertility for wildtype *bam* females over days 1–9 post eclosion (bootstrap 95% confidence interval, effect size). (C) Swarm and Cumming estimation plots showing the total progeny per female for each *W. pipientis* infected line counted on days 1–17 post eclosion. As the fertility assays were performed in batches, each batch is compared to its own uninfected control. *W. pipientis* variants *w*MelPop8X, and *w*MelCS2b were associated with significantly lower fertility over the longer period of days 1–17 post eclosion compared to uninfected controls (bootstrap 95% confidence interval, effect size)

First, to determine if the *w*Mel *bam* hypomorph rescue is a consequence of a general increase in female fertility induced by *W. pipientis*, we assessed the effect of *W. pipientis* variation on the fertility of *w^1118^*; *bam^+^/bam^+^* females of which we have lines infected with 10 different *w*Mel variants of *W. pipientis* as described by Chrostek *et al.* (2013) (Figure 4A).

Here, fertility measured over multiple days reflects progeny produced cumulatively by the female over time, although there could also be variation in development time of the progeny. We first assessed the effect of *W. pipientis* variation on wildtype *bam* female fertility (*w^1118^*; *bam*^+^/*bam*^+^). Over 17 days, we counted the progeny produced by each test female infected individually for each of the 10 *W. pipientis* variants and assessed the mean difference of adult progeny from uninfected control females. We found that none of the *W. pipientis* variants increased female fertility in the wildtype *bam* background, and some lines showed a significant decrease in total progeny per female across all days measured (*w*MelPop8X, and *w*MelCS2b_60) (Figure 4, B and C). Of the lines that showed a negative impact of *W. pipientis* on fertility, this effect was only significant as the female fly aged. On days 1–9 of progeny eclosion, no *W. pipientis* variant had a significantly negative effect on progeny counts, which is notable as these days represent progeny from eggs laid earlier in the test female’s life and thus are more likely to reflect effects of *W. pipientis* variation on fertility in nature (Figure 4B). The variants that showed a significant and large negative effect of *W. pipientis* infection on fertility were both in the *w*MelCS clade (mean difference between −32 and −46 progeny per female for days 1–17 Figure 4C). *w*MelCS variants exhibit higher *W. pipientis* titer in males (Chrostek *et al.* 2013), which if also true in females may negatively impact female fertility if titer gets too high, as this leads apoptosis of infected cells (Chrostek *et al.* 2014).

### All *w*Mel variants rescue *bam* hypomorph female fertility but show a range of effect sizes

**Figure 5:**
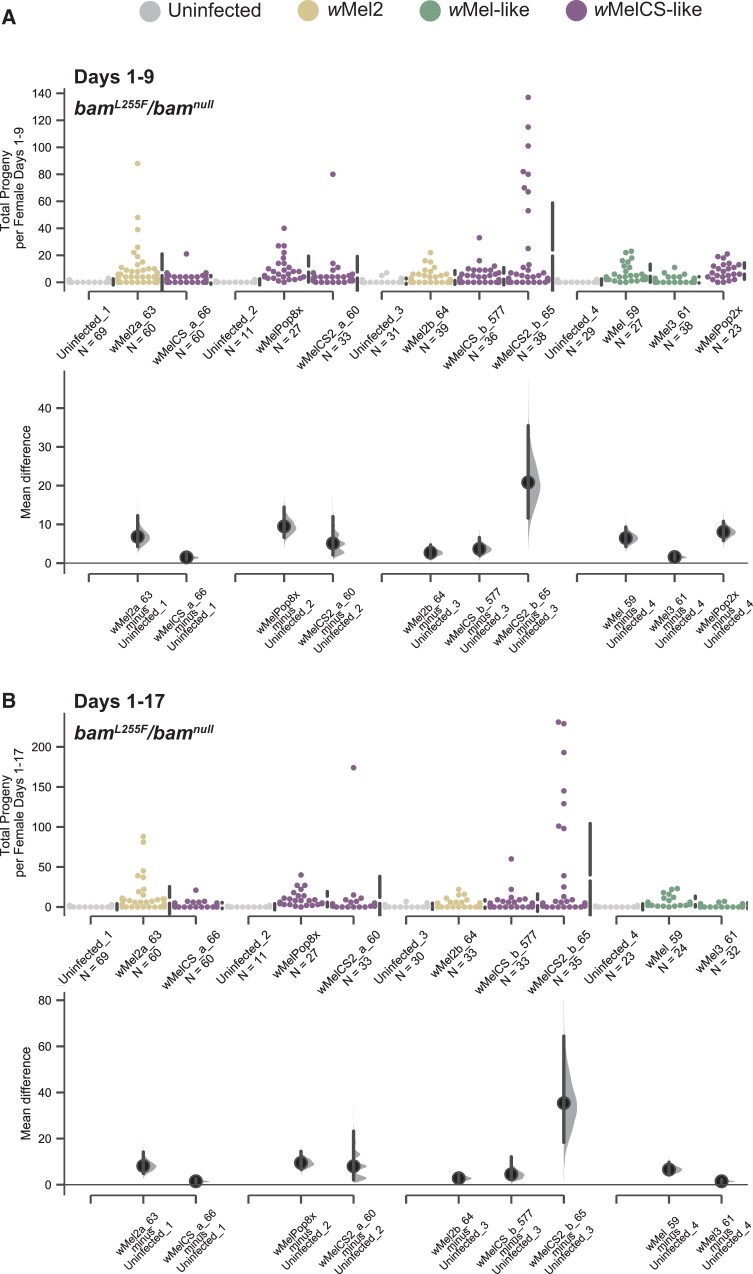
Total progeny per female and mean difference of progeny per female for the *w^1118^*; *bam^L255F^*/*bam^null^* genotype infected with 10 *W. pipientis w*Mel variants compared to uninfected. (A) Swarm and Cumming estimation plots showing the total progeny per female for each *W. pipientis* infected line counted on days 1-9 post eclosion. As the fertility assays were performed in batches, each batch is compared to its own uninfected control. All *W. pipientis* variants were associated with significant increases in female fertility compared to uninfected controls (bootstrap 95% confidence interval, effect size). (B) Swarm and Cumming estimation plots showing the total progeny per female for each *W. pipientis* infected line counted on days 1-17 post eclosion. As the fertility assays were performed in batches, each batch is compared to its own uninfected control. All *W. pipientis* variants were associated with significant increases in female fertility compared to uninfected controls (bootstrap 95% confidence interval, effect size). *w*MelPop2X is not reported for this time frame, as most females died after day 9.

Our results from the *w^1118^*; *bam^+^*/*bam*^+^ fertility assay indicated that none of the *W. pipientis* variants tested have an overall increase in fertility in females (Figure 4). We performed the same fertility assay as described above for the *w^1118^*; *bam^+^*/*bam*^+^ genotype for the *bam^L255F^*/*bam^null^* genotype infected with the same 10 *W. pipientis* variants. Females of the *bam^L255F^*/*bam^null^* genotype exhibit tumorous ovaries and are only weakly fertile (Figures 2B and 3). In contrast to the wildtype *bam* lines, in the *bam^L255F^*/*bam^null^* lines, we found that all variants increased female fertility both in the first week of progeny eclosion and throughout the entire measured timeframe with the exception of *w*MelPop2X, as infected females died before the end of the experiment, thus we only report the first week of progeny production for this variant (Figure 5, A and B). Notably, and some variants had a large effect on female fertility. The variants with the largest effect sizes were in the *w*MelCS-like clade (*w*MelCS2b, *w*MelPop2x, and *w*MelPop8x) (Figure 5, A and B). This fertility rescue is in contrast to the decrease in fertility by the same *W. pipientis* variants in the wildtype *bam* background, especially for *w*MelCS2b which had the largest negative effect on wildtype *bam* fertility, but the largest positive effect on *bam^L255F^*/*bam^null^* fertility (Figure 5, A and B). In all of the lines tested there were many individual females that had no progeny as well as females with varying distributions of progeny. In some of the lines (*w*Mel2a, *w*MelCS2a, and *w*MelCS2b) there were outlier females who had large rescue of fertility, some in the range of what we observe for wildtype *bam* fertility (Figure 4, B and C). However, for most of the individuals tested, fertility was not fully rescued, *e.g.*, restored to wildtype levels (Figures 4, B and C; 5, A and B). Although the largest effect of *W. pipientis* on *bam^L255F^*/*bam^null^* rescue was amongst the *w*MelCS-like variants, there were other notable variants outside of the *w*MelCS clade that showed high increase in female fertility, particularly *w*Mel2a. Overall these data further indicate a genetic interaction between *bam* and *W. pipientis* that is specific from the overall effect of *W. pipientis* on female fertility.

### All *w*Mel variants rescue the ovarian *bam* hypomorph cytological defect but to varying degrees

**Figure 6:**
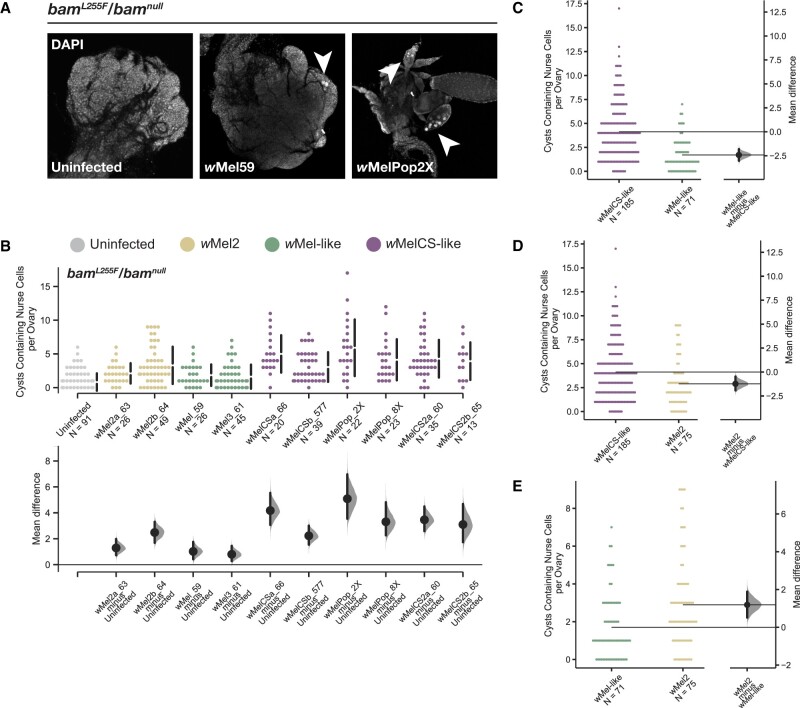
Total cysts containing nurse cells per ovary and mean difference of nurse cell positive cysts for the *w^1118^*; *bam^L255F^*/*bam^null^* genotype infected with 10 *W. pipientis* wMel variants compared to uninfected. (A) Representative images of *bam^L255F^*/*bam^null^* ovaries from uninfected, *w*Mel59, and *w*Melpop2X infected females. Nurse cell positive cysts labeled with arrowheads. (B) Swarm and Cumming estimation plots showing the total cysts containing nurse cells per ovary for each *W. pipientis* infected line assayed on 2–3 days old females. All *W. pipientis* variants are associated with a significant increase in nurse cell positive cysts per ovary (bootstrap 95% confidence interval, effect size). *W. pipientis* variants in the *w*MelCS-like clade have the highest effect on nurse cell positive cysts. (C–E) Swarm and Gardner-Altman plots showing pairwise comparisons of nurse cell positive cyst counts pooled together for each *w*Mel clade. *w*MelCS-like variants exhibit higher effects on nurse cell positive cysts compared to *w*Mel2 and *w*Mel-like variants (95% confidence interval, effect size), and *w*Mel2 variants exhibit a higher effect on nurse cell positive cysts than *w*Mel-like (95% confidence interval, effect size).

**Figure 7:**
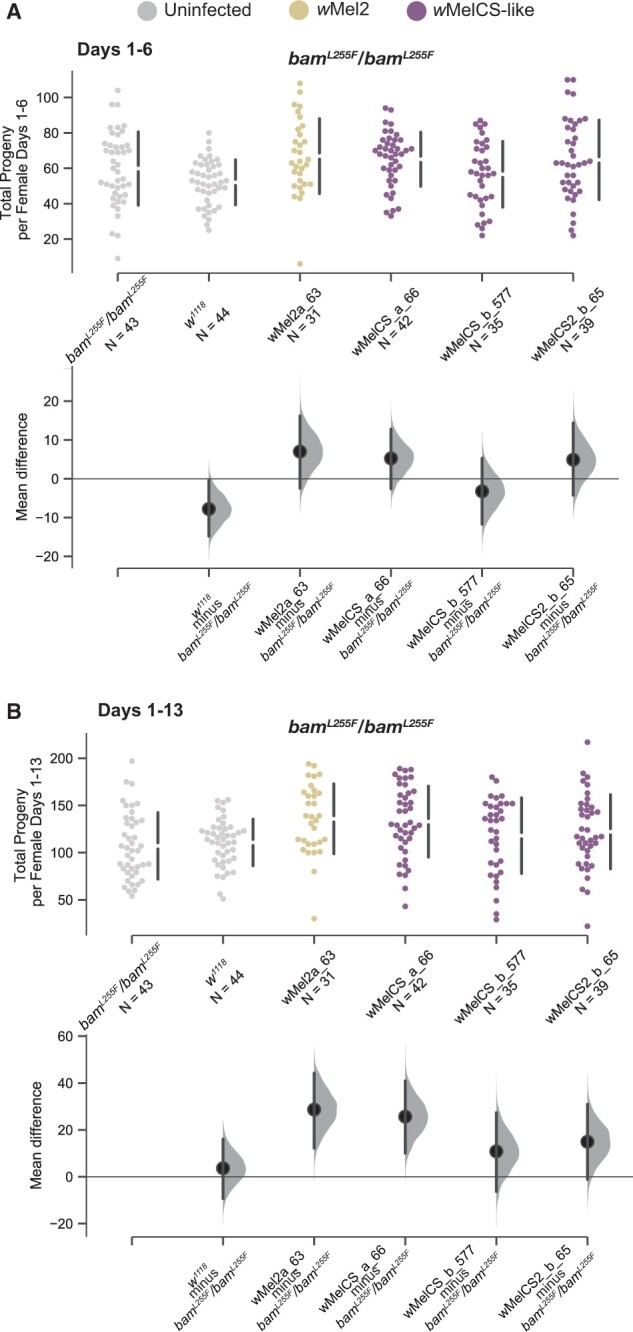
Total progeny per female and mean difference of progeny per female for the *w^1118^*; *bam^L255F^*/*bam^L255F^* genotype infected with four representative *W. pipientis* variants compared to the uninfected control. (A) Swarm and Cumming estimation plots showing the effect of *Wolbachia* variants on *bam^L255F^*/*bam^L255F^* female fertility over days 1–6 of eclosion. We included uninfected *bam*^+^/*bam*^+^ females as well to compare *bam^L255F^*/*bam^L255F^* fertility to *bam*^+^/*bam*^+^ fertility. *W. pipientis* variants did not have differential effects on female *bam^L255F^*/*bam^L255F^* fertility during this time frame. The *bam^L255F^*/*bam^L255F^* genotype regardless of *W. pipientis* infection showed a wider range of progeny compared to *bam*^+^/*bam*^+^ females, and uninfected *bam*^+^/*bam*^+^ mean fertility was significantly lower than uninfected *bam^L255F^*/*bam^L255F^* mean fertility (95% confidence interval, effect size). (B) Swarm and Cumming estimation plots showing the effect of *W. pipientis* variants on *bam^L255F^*/*bam^L255F^* female fertility over days 1–13 of eclosion. *W. pipientis* variants had differential effects on female *bam^L255F^*/*bam^L255F^* fertility. No variants had a negative effect on fertility. *Wolbachia* variants wMel2a_63, wMelCS_a_66 showed significant positive effects on fertility. The *bam^L255F^*/*bam^L255F^* genotype regardless of *W. pipientis* infection showed a wider range of progeny compared to *bam*^+^/*bam*^+^ females, but uninfected *bam*^+^/*bam*^+^ and uninfected *bam^L255F^*/*bam^L255F^* mean fertility was not significantly different.

Although we wanted to assess the rescue of the *bam* hypomorphic fertility phenotype by *W. pipientis* because we can also compare this to the effect of *W. pipientis* on wildtype *bam* fertility, we also wanted to know if genetic variation in *W. pipientis* affected the rescue of the *bam* hypomorphic cytological phenotype. Because fertility is affected by environmental and genetic differences that act in later developmental stages outside of *bam’*s function early in gametogenesis, we also measured the effect of *W. pipientis* variation on the rescue of the cytological *bam* mutant phenotype. Since the partial loss of function of *bam* results in over-proliferation of GSCs and reduced differentiation, we can quantify *bam* function by assaying for differentiated germ cells. The cytological *bam* hypomorphic phenotype in females consists of tumorous ovarioles filled with GSC-like cells, with few properly developing egg chambers (Figure 6A). Properly developing egg chambers contain polyploid nurse cells, which feature large, easily identifiable nuclei (Figure 6A, arrowheads). We counted the number of nurse cell positive egg chambers as a measure of GSC daughter differentiation and thus *bam* function. We found that all of the *W. pipientis* variants tested significantly increased the number of nurse cell positive egg chambers, and that *W. pipientis* variants in the *w*MelCS-like clade showed highest numbers of nurse cell positive egg chambers (Figure 7B). We then pooled together the data individually for *w*Mel-like, *w*Mel2-like, and *w*MelCS-like and asked if there was a difference in mean number of cysts containing nurse cells per ovary between each clade (Figure 6, C and D). We found that *w*MelCS-like variants had a significantly higher effect size than *w*Mel-like and *w*Mel2-like (Figure 6, C and D), and that wMel2-like had a higher effect than *w*Mel-like (Figure 6E). These results are consistent with the fertility assay results for the *bam^L255F^*/*bam*^null^ hypomorph genotype, however we observed a more obvious trend of higher *bam* rescue by the *w*MelCS-like variants in this assay. All of the *W. pipientis* variants with the highest effect size of increased *bam* function are in the *w*MelCS-like clade. It is unclear whether this higher rescue is due to titer or another genetic difference between these clades, but these data indicate that genetic variation in *W. pipientis* impacts the *bam* rescue phenotype.

To ask if the nurse cell positive egg chambers resulted in oocytes capable of development if fertilized, and therefore represent properly differentiated GSC daughters, we picked two representative *W. pipientis* variants of high and low rescue and counted the total number of eggs laid per female over days 1–3. In *w^1118^*; *bam*^+^/*bam*^+^ females, neither *W. pipientis* variant affected the number of eggs laid per female (Supplementary Figure S1A). In contrast to the *w^1118^*; *bam*^+^/*bam*^+^ females and consistent with the nurse cell assay, we observed few eggs laid by uninfected *w^1118^*; *bam^L255F^*/*bam^null^* females, with significantly more eggs produced by the lower rescuing *W. pipientis w*Mel59, and the highest number of eggs laid by females infected with the high rescuing line, *w*MelCSa_66 (Supplementary Figure S1B).

### Homozygous *bam^L255F^* females exhibit more variable fertility which is not negatively affected by *W. pipientis*

In light of the variable effect of infection by some *W. pipientis* variants on wildtype *bam* fertility after day 9, and that the uninfected *bam^L255F^*/*bam^L255F^* females did not exhibit a statistically significant fertility defect (Figures 2G;4, B and C), we asked if infection by a subset of the *W. pipientis* variants we assessed in the wildtype *bam* background had any effect on the fertility of *bam^L255F^*/*bam^L255F^* females. We chose three *W. pipientis* variants that did not have a significant effect on wildtype *bam* female fertility for the entire time frame (*w*Mel2a_63, *w*MelCSa_66, and *w*MelCSb_577 Figure 7, A and B), and one that had a significant negative effect on female fertility in the second half of the experiment (*w*MelCS2b, Figure 7B). We measured fertility of homozygous females infected with *W. pipientis* variants as previously described. In both *bam*^+^/*bam*^+^ and *bam^L255F^*/*bam^L255F^* backgrounds, no *w*Mel variants affected fertility during the first half of the experiment (Figures 4B and 7A). However, in contrast to the *bam*^+^/*bam*^+^ background where *w*MelCS2b negatively affected female fertility across all days measured, we found that in the *bam^L255F^*/*bam^L255F^* background *w*MelCS2b did not have a significant effect on female fertility (Figure 7B). In addition, in contrast to our findings in the *bam*^+^/*bam*^+^ background that *w*Mel2a_63 and *w*MelCSa_66 did not significantly affect female fertility, we found that in the *bam^L255F^*/*bam^L255F^* background both of these variants had a significantly positive effect on female fertility across all days measured (Figure 7B).

Overall, we observed a more positive effect of *W. pipientis* on female fertility in the *bam^L255F^*/*bam^L255F^* background than in the *bam*^+^/*bam*^+^ background. As the differential effects of *W. pipientis* on female fertility in both *bam* backgrounds were only in older females, this may be related to the general decline in fecundity as the female ages. Although we did not observe a tumorous ovarian phenotype in the *bam^L255F^*/*bam^L255F^* females, it is possible that *W. pipientis* is interacting with a subtle *bam* phenotype that becomes more apparent as the fly ages and oogenesis declines. This further indicates that there is an effect of *bam* genotype and *W. pipientis* genotype on the interaction between *W. pipientis* and female fertility, and that *W. pipientis* does not just generally increase female fertility in a wildtype *bam* background. In addition, this interaction does not require the *bam* tumor phenotype.

Interestingly, during days 1–6 uninfected wildtype *bam* females had significantly fewer progeny than uninfected homozygous *bam^L255F^* females (Figure 7A). We also observed that the total progeny per female of the homozygous *bam^L255F^* genotype had a wider range than wildtype, which was not impacted by *W. pipientis* and is therefore likely a subtle *bam* mutant phenotype (Figure 7A). As *bam* regulates GSC daughter differentiation, its mis-regulation may increase or decrease fertility.

## Discussion

### Revisiting the phenotypes of classic *bam* alleles using CRISPR/Cas9 in a controlled genetic background

We previously reported that infection with *w*Mel rescued the fertility defect of *bam^BW^/bam^Δ59^* hypomorphic mutant females (Flores *et al.* 2015). We sought to better understand this interaction for multiple reasons. The first reason is that we hypothesize that an interaction between *W. pipientis* and *bam* may be driving the adaptive sequence divergence of *bam*. However, in order to understand if and how this interaction could be adaptive, we need to understand the nature of the manipulation of *bam* function by *W. pipientis*. Second, *W. pipientis* is of immense interest as a potential means to control vectors of some human diseases (Moreira *et al.* 2009).

One such way to find the *W. pipientis* loci necessary for the interaction between bacteria and the host would be to systemically mutagenize the bacteria and then screen for variants that enhance or reduce the phenotype of interest. However, *W. pipientis* are obligate endosymbionts that cannot be cultured, and so are currently not amenable to such genetic manipulation. While some studies have identified *W. pipientis* loci that interact with *D. melanogaster* by transgenically expressing *W. pipientis* loci in *D. melanogaster*, this requires identifying candidate *W. pipientis* loci that affect the host phenotype of interest (Ote *et al.* 2016; Le Page *et al.* 2017). An alternative method to identify candidate *W. pipientis* loci that affect the host phenotype is to use genetic variation in *W. pipientis* to test for differences in the effect of *W. pipientis* variants on the host phenotype.

Chrostek *et al.* (2013) generated isogenic *Drosophila* lines infected with previously described and genetically distinct *w*Mel variants of *W. pipientis*. The original *bam^BW^* mutant was not in this host genetic background and thus we would not be able to conclude that any differential interaction between *bam* and these variants was due to *W. pipientis* variation and not host genetic variation. Therefore, we used CRISPR/Cas9 to create the same amino acid mutant present in the original *bam^BW^* hypomorph allele in the *w^1118^* isogenic background. To ensure we had the tools to assess the *W. pipientis* rescue phenotype as previously described, we also generated a new *bam* null allele using CRISPR/Cas9 in the same *w^1118^* genetic background.

We have now confirmed that these two new alleles behave similarly to the alleles we previously used to document the interaction between *w*Mel and *bam*. Of note, our *bam^L255F^/bam^null^* hypomorph exhibits a stronger fertility defect and GSC daughter differentiation defect in comparison to our previous findings in the *bam^BW^/bam^Δ59^* hypomorph (Flores *et al.* 2015). Here, for uninfected *bam^L255F^/bam^null^* females, we observed a mean progeny per female of ∼2 and we observed a mean of ∼2 cysts containing nurse cells per ovary (Figures 5 and 6). For the *bam^BW^/bam^Δ59^* hypomorph, we previously observed a mean progeny per female of ∼40 and ∼2 nurse cell containing cysts per ovariole (Flores *et al.* 2015). Notably, each ovary contains 15–20 ovarioles, so 2 nurse cell containing cysts per ovariole would correspond to ∼30–40 nurse cell containing cysts per ovary. Therefore, *w*Mel not only rescues the mild GSC daughter differentiation defect in the original *bam^BW^/bam^Δ59^* hypomorph, but *w*Mel and the other nine variants tested here also rescue the stronger *bam* GSC daughter differentiation defect we observe in the *bam^L255F^/bam^null^* hypomorph. This is especially notable, since *w*Mel does not rescue fertility or GSC daughter differentiation in *bam* null mutants studied by Flores *et al.* (2015). Therefore, the *w*Mel rescue of the *bam* mutant phenotype requires some functional *bam* gene product, but is also robust to a more severe fertility and differentiation defect. The difference in severity between the two *bam* hypomorphic genotypes also highlights the importance in controlling genetic background to rigorously assess fecundity phenotypes.

There have been a handful of studies assessing the phenotypes of diverse *W. pipientis* in isogenic backgrounds (Chrostek *et al.* 2013, 2014; Chrostek and Teixeira 2018; Gruntenko *et al.* 2019), however, there have been no documented studies of interactions between *Drosophila* mutants and *W. pipientis* in these backgrounds. Recent work on the interaction between *W. pipientis* and *Sex lethal* in the female germline showed that *W. pipientis* rescues the loss of GSCs in some *Sxl* mutants through a *nanos* dependent interaction with the *W. pipientis* protein TomO (Ote *et al.* 2016). However, when researchers transgenically expressed *TomO* in *Sxl* hypomorphic ovaries, the GSC number was rescued, but fertility was not. This result indicated that there are other mechanisms *W. pipientis* is using to fully rescue GSC number and fertility in *Sxl* mutants. We believe the strategy we describe here could be used to further define genetic interactions such as the *Sxl* and *W. pipientis* interaction (Starr and Cline 2002). As we and others have also had success with CRISPR/Cas9 in non-*melanogaster* species, we believe this type of systematic analysis could be extended to assess interactions of host genes and *W. pipientis* in other species outside of *Drosophila melanogaster*.

### *w*Mel *W. pipientis* variants do not broadly increase fertility in the *w*^1118^ genetic background

Due to the shared *w*^1118^ genetic background of the *W. pipientis* infected lines, we were able to rigorously assess the effect of different *W. pipientis* variants on wildtype *bam* female fertility. In addressing if and how *W. pipientis* may drive the adaptive evolution of *bam*, it was important for us to know if *W. pipientis* has an effect on fertility in a wildtype *bam* background. Here, we are not measuring specifically how *W. pipientis* is modulating a functional *bam* allele, as fertility is affected by many loci, but if we observed a large effect of a particular *W. pipientis* variant on fertility, this would motivate further experiments to assess functional *bam* activity in lines infected with that *W. pipientis* variant. We observed no line with increased fertility for any of the *W. pipientis* variants, indicating that *W. pipientis* is not generally increasing fertility through *bam* or another pathway in this genetic background. However, we also observed that some *W. pipientis* variants had a negative effect on female fertility as the females aged. We cannot distinguish whether this effect is due to *W. pipientis* mis-regulating the germline or some other developmental consequence of its infection. In fact, the lines with the highest reported titers in males showed the largest negative impact on female fertility (Chrostek *et al.* 2013). In addition, Serga *et al.* (2014) reported lower fecundity for females from a natural population in Uman, Ukraine infected with *w*MelCS compared to females infected with *w*Mel. These observations highlight the complexity of the interaction between *W. pipientis* and *Drosophila*, where depending on the genetic background of the host, the fitness effect of *W. pipientis* on a phenotype may vary. This effect could further vary based on aspects of the host’s environment where the manipulation of fertility by *W. pipientis* may not always be beneficial. Here, we may expect the host and microbe to evolve ways of evading each other, which may become more or less apparent in different genetic backgrounds. Of note, our observations were made under laboratory conditions in a highly inbred line, and so we cannot be sure these *W. pipientis* variants impart the same effects on female fertility in natural populations.

### *w*Mel *W. pipientis* variants across three clades genetically interact with *bam*

We previously showed that a single *w*Mel variants partially rescued the fertility and GSC daughter differentiation defect of the *bam^BW^/bam^Δ59^* hypomorph. Here, we asked if there was variation in the *bam* rescue phenotype among *W. pipientis variants*. The 10 variants we used have been fully sequenced and differ by varying degrees of sequence divergence. All 10 *W. pipientis* variants of the three clades tested (III, VI, and VIII; see Figure 4A) rescued the fertility defect and the cytological defect of the *bam^L255F^*/*bam^null^* hypomorph. Therefore, none of the variants tested contained genetic variation that suppressed the interaction between *bam* and *W. pipientis*. We did find that in both the counts of nurse cell positive egg chambers and the fertility assays, *w*MelCS-like *W. pipientis* variants (clade VI) showed the highest rescue (Figures 5 and 6). This pattern was the clearest in the counts of nurse cell positive egg chambers as expected, since the presence of nurse cell positive egg chambers reflects GSC daughter differentiation, a phenotype that is a more direct output of *bam* activity. It is likely that the higher level of variability in the fertility assays is also due to the variable effect the *W. pipientis* variants have on other stages of development post GSC daughter differentiation. However, we feel it is important to also measure the effect of *W. pipientis* on fertility, since we cannot assess the count of nurse cell positive egg chambers for *bam* alleles that do not show a tumorous mutant phenotype, and it gives us insight into how *W. pipientis* infection could be adaptive. For example, would there be a fitness tradeoff between a high fecundity (GSC daughter differentiation) rescue and a low fertility (adult progeny) rescue? Measuring the impact of *W. pipientis* on multiple stages of oogenesis and reproduction therefore gives us insight into any further complexities in the interaction between GSC genes and *W. pipientis*.

We found that the *w*MelCS-like variants had the highest rescue effect on *bam* function, another example of a *W. pipientis* induced *D. melanogaster* fitness phenotype of which *w*MelCS-like variants exhibit the highest effects. The *w*MelCS-like variants tested here also confer the highest levels of viral resistance to *D. melanogaster* males (Chrostek *et al.* 2013). In addition, *w*MelCS-like variants have a stronger effect on the thermal preference of the *D. melanogaster* host, with *w*MelCS-like variants conferring preference for cooler temperatures compared to *w*Mel-like and uninfected *D. melanogaster* (Truitt *et al.* 2019)*.* Another study showed that *w*MelCS-like variants increased stress resistance in *D. melanogaster*, and no observed effect from *w*Mel-like variants (Gruntenko *et al.* 2017).*w*MelPop is a virulent derivative of *w*MelCSb and characterized by uncontrolled proliferation and titer caused by increased copy number of the Octomom locus. *w*MelPop2X and *w*MelPop8X exhibit 2X copies of the Octomom locus and anywhere from 4X to 8X copies, respectively (Chrostek and Teixeira 2018). The high rescue of both the cytological and fertility defect of the *bam^L255F^*/*bam^null^* by *w*MelPop2X is suggestive that titer and possibly other phenotypes that increase interactions with the host are contributing to the rescue of *bam*. We observed lower rescue for *w*MelPop8X, indicating that the increasing copy number of the Octomom locus that causes apoptosis and early death likely negatively affects host fertility (Zhukova and Kiseleva 2012; Chrostek and Teixeira 2018). *w*MelPop2X is closely related to *w*MelCSb, with the only variation between them being a synonymous SNP and the amplification of the octomom locus which contains *W. pipientis* loci predicted to be involved with nucleic acid binding, and thus likely how it increases its titer, as well as proteins with predicted homology to eukaryotic domains (Chrostek and Teixeira 2018). These loci may then increase the interaction of *W. pipientis* and its host (López-Madrigal and Duarte 2020). A natural next step would be to determine the *W. pipientis* factors that manipulate GSC daughter differentiation.

### Possible adaptive interactions between *W. pipientis* and *bam*

It is well established that disrupting *bam* function negatively effects fertility (McKearin and Spradling 1990; Ohlstein and McKearin 1997; Lavoie *et al.* 1999; Flores *et al.* 2015), and *bam^L255F^*/*bam^null^* females are almost completely sterile. However, we do not predict such deleterious alleles to reach high frequency in natural populations. In fact, we have not found this nucleotide variant segregating in any of the natural populations of *D. melanogaster* that have been sampled in the *Drosophila* genome Nexus (Lack *et al.* 2015). Thus, while studying this mutant is effective in further refining how *bam* and *W. pipientis* interact, we cannot conclude that this type of interaction occurs in natural populations. However, given the severity of the mutant and strength of *W. pipientis’*s rescue, one hypothesis is that if *W. pipientis* is increasing GSC daughter differentiation when it is not favorable for the host, the host would evolve a way to evade *W. pipientis’*s manipulation of this pathway. In the case of our lab generated mutant, it is possible that when *bam* is not fully functional that reproduction is more sensitive to manipulation by *W. pipientis*. *W. pipientis* have been documented to respond to changes in the host environment, as *W. pipientis* gene expression is affected by host age and sex, and as *W. pipientis* transmission requires functional oogenesis, it reasonable to hypothesize that *W. pipientis* is sensitive to changes in gametogenesis (Rice *et al.* 2017; Newton and Sheehan 2018; Russell *et al.* 2020).

An additional observation we made is that *bam^L255F^*/*bam^L255F^* females show a broader range of adult progeny per female compared to wildtype *bam* females regardless of *W. pipientis* infection status. The individual *bam^L255F^*/*bam^L255F^* females exhibit both higher and lower than average fertility, indicating that mis-regulating *bam's* differentiation function could both increase and decrease fertility. This phenotype is worth further investigation, since although *bam* shows a signature of positive selection, we do not know specifically what aspect of *bam* function is adaptive. If variation in *bam* function can both increase and decrease mean fertility, and *W. pipientis* has a generally positive effect on fertility of *bam^L255F^*/*bam^L255F^* females, this is further evidence that *W. pipientis* may be able to manipulate *bam* in order to increase oogenesis for its own benefit, and that genetic variation at *bam* could affect the regulation of oogenesis. Therefore, if *W. pipientis* increased the rate of oogenesis to ensure its own transmission and this was not favorable for the host, *bam* may be in conflict with *W. pipientis* to regulate oogenesis in a favorable manner for the host. Here we see that although the average female fertility of uninfected *bam^L255F^*/*bam^L255F^* mutants is not significantly different from wildtype, there is a wider distribution of individual female fertility, indicating that the *bam* hypomorphic phenotype is an increased variance in fertility (Figure 7). Since these females are in a common genetic background and we do not expect genetic variation between individual females, the *bam*-mediated mis-regulation of differentiation may set off a cascade of other genetic mis-regulation that results in higher or lower fertility over time. We see that infection by some *W. pipientis* variants in this background increases female fertility, indicating that perhaps *W. pipientis* can restore the mis-regulation of GSC daughter differentiation, and even increase reproductive output through this mechanism. It would be interesting to use this genotype for future experiments to explore the possibility that this *bam* mutation may disrupt the mechanism *D. melanogaster* evolved to evade *W. pipientis’*s effect on differentiation.

An interesting question that remains is how *W. pipientis* have evolved in their interaction with GSC genes, including *bam*. While *w*MelCS-like and *w*Mel-like variants share a most recent common ancestor about 8000 years ago, *w*MelCS-like variants were recently (∼2000 years ago) replaced by *w*Mel-like variants in natural populations of *D. melanogaster* (Riegler *et al.* 2005; Richardson *et al.* 2012). However, this replacement has not been complete, and there are global populations still infected with *w*MelCS variants (Riegler *et al.* 2005; Nunes *et al.* 2008). Interestingly we find that the *w*MelCS variants we assayed show higher rescue of *bam^L255F^*/*bam^null^* female fertility and nurse cells. If *bam* and *W. pipientis* are evolving in an arms race, it could be that the evolutionarily more recent *w*Mel-like variants have not evolved the same level of interaction with *bam* as *w*MelCS has. Future work to investigate these dynamics should include sampling populations that are still infected with *w*MelCS as well as those infected with *w*Mel and asking if there is any evidence of genetic differentiation at *bam*. Some populations have been identified that are still infected with *w*MelCS, such as a natural population of *D. melanogaster* from Uman, Ukraine that has been infected with both *w*Mel and *w*MelCS and has been monitored yearly for infection frequency, some additional Paleartic populations, and a population in the Netherlands (Early and Clark 2013; Bykov *et al.* 2019; Serga *et al.* 2021).

In addition, we could utilize existing *W. pipientis* sequence variation from natural populations to ask if there is any evidence of associations between *bam* variation and *W. pipientis* variation. If we were to sample populations that are differentially infected with *w*Mel and *w*MelCS to assess genetic differentiation at *bam* as discussed above, we could also do the same for *W. pipientis* variants. One caveat being that if we did not observe genetic signatures of adaptive evolution in *W. pipientis* loci, this does not mean that *W. pipientis* and *bam* are not coevolving, as we do not know the true infection history of a *Drosophila* population with *W. pipientis* and thus which *W. pipientis* variant may have been in conflict with *bam.* In addition, there may be general *W. pipientis* functions that are interacting with *bam* and not a single locus (*e.g.*, loci that regulate titer).

Further work should focus on determining the *W. pipientis* loci contributing to the different magnitude of *bam* rescue. However, as has been previously pointed out by Chrostek *et al.* (2013), between the *w*Mel clades and *w*MelCS clades there are eight indels and 108 SNPs, including differences in the coding sequence of 58 genes. So further work would have to be done to narrow down which variants contribute to the degree of rescue. A next step could be moving out to more divergent *W. pipientis wMel* variants [for example, *w*Au that infects *D. simulans* (Miller and Riegler 2006)] to ask how recently the interaction with *bam* evolved. To complement this, it would be beneficial to perform these same rescue experiments with *bam* hypomorphs in *D. simulans*.

## Data availability

Fly lines and plasmids used in this study are available upon request. The authors affirm that all data necessary for confirming the conclusions of the article are present within the article, figures, and tables.

Supplementary material is available at *G3* online.

## Supplementary Material

jkab312_Supplementary_Data

## References

[jkab312-B1] Arnold PA , Levin SC, Stevanovic AL, Johnson KN. 2019. *Drosophila melanogaster* infected with *Wolbachia* strain wMelCS prefer cooler temperatures. Ecol Entomol. 44:287–290. doi:10.1111/een.12696.

[jkab312-B2] Aruna S , Flores HA, Barbash DA. 2009. Reduced fertility of *Drosophila melanogaster* *hybrid male rescue* (*Hmr*) mutant females is partially complemented by *Hmr* orthologs from sibling species. Genetics. 181:1437–1450. 10.1534/genetics.108.100057 19153254 19153254 PMC2666511

[jkab312-B3] Bailly-Bechet M , Martins-Simões P, Szöllosi GJ, Mialdea G, Sagot MF, et al 2017. How long does *Wolbachia* remain on board? Mol Biol Evol. 34:1183–1193. doi:10.1093/molbev/msx073. 28201740 10.1093/molbev/msx073

[jkab312-B4] Bauer DuMont VL , Flores HA, Wright MH, Aquadro CF. 2007. Recurrent positive selection at *bgcn*, a key determinant of germ line differentiation, does not appear to be driven by simple coevolution with its partner protein Bam. Mol Biol Evol. 24:182–191. doi:10.1093/molbev/msl141. 17056645 10.1093/molbev/msl141

[jkab312-B5] Beckmann JF , Ronau JA, Hochstrasser M. 2017. A *Wolbachia* deubiquitylating enzyme induces cytoplasmic incompatibility. Nat Microbiol. 2:17007.doi:10.1038/nmicrobiol.2017.7. 28248294 10.1038/nmicrobiol.2017.7 PMC5336136

[jkab312-B6] Bykov RA , Yudina MA, Gruntenko NE, Zakharov IK, Voloshina MA, et al 2019. Prevalence and genetic diversity of *Wolbachia* endosymbiont and mtDNA in Palearctic populations of *Drosophila melanogaster*. BMC Evol Biol. 19:48.doi:10.1186/s12862-019-1372-9. 30813886 10.1186/s12862-019-1372-9 PMC6391860

[jkab312-B7] Choi JY , Aquadro CF. 2014. The coevolutionary period of *Wolbachia pipientis* infecting *Drosophila ananassae* and its impact on the evolution of the host germline stem cell regulating genes. Mol Biol Evol. 31:2457–2471. doi:10.1093/molbev/msu204. 24974378 10.1093/molbev/msu204 PMC4137719

[jkab312-B8] Chrostek E , Marialva MSP, Esteves SS, Weinert LA, Martinez J, et al 2013. *Wolbachia* variants induce differential protection to viruses in *Drosophila melanogaster*: a phenotypic and phylogenomic analysis. PLoS Genet. 9:e1003896.doi:10.1371/journal.pgen.1003896. 24348259 10.1371/journal.pgen.1003896 PMC3861217

[jkab312-B9] Chrostek E , Marialva MSP, Yamada R, O'Neill SL, Teixeira L. 2014. High anti-viral protection without immune upregulation after interspecies *Wolbachia* transfer. PLoS One. 9:e99025.doi:10.1371/journal.pone.0099025. 24911519 10.1371/journal.pone.0099025 PMC4049622

[jkab312-B10] Chrostek E , Teixeira L. 2018. Within host selection for faster replicating bacterial symbionts. PLoS One. 13:e0191530. doi:10.1371/journal.pone.0191530. 29346449 10.1371/journal.pone.0191530 PMC5773213

[jkab312-B11] Dedeine F , Vavre F, Fleury F, Loppin B, Hochberg ME, et al 2001. Removing symbiotic *Wolbachia* bacteria specifically inhibits oogenesis in a parasitic wasp. Proc Natl Acad Sci USA. 98:6247–6252. doi:10.1073/pnas.1013042 11353833 10.1073/pnas.101304298 PMC33453

[jkab312-B12] Deehan M , Lin W, Blum B, Emili A, Frydman H. 2021. Intracellular density of *Wolbachia* is mediated by host autophagy and the bacterial cytoplasmic incompatibility gene *cifB* in a cell type-dependent manner in *Drosophila melanogaster*. mBio. 12:1–19. doi:10.1128/mBio.02205-20. 10.1128/mBio.02205-20 PMC7844536 33436431

[jkab312-B13] Dutra HLC , Rocha MN, Dias FBS, Mansur SB, Caragata EP, et al 2016. *Wolbachia* blocks currently circulating zika virus isolates in Brazilian *Aedes aegypti* mosquitoes. Cell Host Microbe. 19:771–774. doi:10.1016/j.chom.2016.04.021. 27156023 10.1016/j.chom.2016.04.021 PMC4906366

[jkab312-B14] Early AM , Clark AG. 2013. Monophyly of *Wolbachia pipientis* genomes within *Drosophila melanogaster*: geographic structuring, titre variation and host effects across five populations. Mol Ecol. 22:5765–5778. doi:10.1111/mec.12530. 24118111 10.1111/mec.12530 PMC4005148

[jkab312-B15] Flores HA , Bubnell JE, Aquadro CF, Barbash DA. 2015. The *Drosophila* *bag of marbles* gene interacts genetically with *Wolbachia* and shows female-specific effects of divergence. PLoS Genet. 11:e1005453. doi:10.1371/journal.pgen.1005453. 26291077 10.1371/journal.pgen.1005453 PMC4546362

[jkab312-B16] Gratz SJ , Ukken FP, Rubinstein CD, Thiede G, Donohue LK, et al 2014. Highly specific and efficient CRISPR/Cas9-catalyzed homology-directed repair in *Drosophila*. Genetics. 196:961–971. doi:10.1534/genetics.113.160713. 24478335 10.1534/genetics.113.160713 PMC3982687

[jkab312-B17] Gruntenko NE , Ilinsky YY, Adonyeva NV, Burdina EV, Bykov RA, et al 2017. Various *Wolbachia* genotypes differently influence host *Drosophila* dopamine metabolism and survival under heat stress conditions. BMC Evol Biol. 17:252.doi:10.1186/s12862-017-1104-y. 29297293 10.1186/s12862-017-1104-y PMC5751659

[jkab312-B18] Gruntenko NE , Karpova EK, Adonyeva NV, Andreenkova OV, Burdina EV, Ilinsky YY, et al 2019. *Drosophila* female fertility and juvenile hormone metabolism depends on the type of *Wolbachia* infection. J Exp Biol. 222:jeb195347.doi:10.1242/jeb.195347. 30679245 10.1242/jeb.195347

[jkab312-B19] Hague MTJ , Caldwell CN, Cooper BS. 2020. Pervasive effects of *Wolbachia* on host temperature preference. MBio. 11:e01768-20. doi:10.1128/mBio.01768-20. 33024036 10.1128/mBio.01768-20 PMC7542361

[jkab312-B20] Ho J , Tumkaya T, Aryal S, Choi H, Claridge-Chang A. 2019. Moving beyond P values: data analysis with estimation graphics. Nat Methods. 16:565–566. doi:10.1038/s41592-019-0470-3. 31217592 10.1038/s41592-019-0470-3

[jkab312-B21] Hoffmann AA , Montgomery BL, Popovici J, Iturbe-Ormaetxe I, Johnson PH, et al 2011. Successful establishment of *Wolbachia* in Aedes populations to suppress dengue transmission. Nature. 476:454–457. doi:10.1038/nature10356. 21866160 10.1038/nature10356

[jkab312-B22] Insco ML , Bailey AS, Kim J, Olivares GH, Wapinski OL, et al 2012. A self-limiting switch based on translational control regulates the transition from proliferation to differentiation in an adult stem cell lineage. Cell Stem Cell. 11:689–700. doi:10.1016/j.stem.2012.08.012. 23122292 10.1016/j.stem.2012.08.012 PMC3833810

[jkab312-B23] Lack JB , Cardeno CM, Crepeau MW, Taylor W, Corbett-Detig RB, et al 2015. The *Drosophila* genome nexus: a population genomic resource of 623 *Drosophila melanogaster* genomes, including 197 from a single ancestral range population. Genetics. 1229–1241. doi:10.1534/genetics.115.174664. 10.1534/genetics.115.174664 PMC4391556 25631317

[jkab312-B24] Lavoie CA , Ohlstein B, McKearin DM. 1999. Localization and function of Bam protein require the *benign gonial cell neoplasm* gene product. Dev Biol. 405–413. 212:doi:10.1006/dbio.1999.9346. 10433830 10.1006/dbio.1999.9346

[jkab312-B25] Lindsey ARI. 2020. Sensing, signaling, and secretion: a review and analysis of systems for regulating host interaction in *Wolbachia*. Genes (Basel). 11:1–21. doi:10.3390/genes11070813. 10.3390/genes11070813 PMC7397232 32708808

[jkab312-B26] López-Madrigal S , Duarte EH. 2020. Titer regulation in arthropod-*Wolbachia* symbioses. FEMS Microbiol Lett. 366:fnz232. doi:10.1093/femsle/fnz232. 10.1093/femsle/fnz232 31750894

[jkab312-B27] McKearin DM , Spradling AC. 1990. *Bag-of-marbles*: a *Drosophila* gene required to initiate both male and female gametogenesis. Genes Dev. 4:2242–2251. doi:10.1101/gad.4.12b.2242. 2279698 10.1101/gad.4.12b.2242

[jkab312-B28] Miller WJ , Riegler M. 2006. Evolutionary dynamics of wAu-like *Wolbachia* variants in neotropical *Drosophila* spp. Appl Environ Microbiol. 72:826–835. doi:10.1128/AEM.72.1.826-835.2006. 16391124 10.1128/AEM.72.1.826-835.2006 PMC1352291

[jkab312-B29] Moreira LA , Iturbe-Ormaetxe I, Jeffery JA, Lu G, Pyke AT, et al 2009. A *Wolbachia* symbiont in *Aedes aegypti* limits infection with dengue, Chikungunya, and Plasmodium. . Cell. 139:1268–1278. doi:10.1016/j.cell.2009.11.042. 20064373 10.1016/j.cell.2009.11.042

[jkab312-B30] Newton ILG , Sheehan KB. 2018. Gateway entry vector library of *Wolbachia pipientis* candidate effectors from strain *w* Mel. Microbiol Resour Announc. 7:e00806-18. doi:10.1128/mra.00806-18. 30533608 10.1128/MRA.00806-18 PMC6211342

[jkab312-B31] Nunes MDS , Nolte V, Schlötterer C. 2008. Nonrandom *Wolbachia* infection status of *Drosophila melanogaster* strains with different mtDNA haplotypes. Mol Biol Evol. 25:2493–2498. doi:10.1093/molbev/msn199. 18780877 10.1093/molbev/msn199 PMC2568037

[jkab312-B32] Ohlstein B , Lavoie CA, Vef O, Gateff E, Mckearin DM. 2000. The *Drosophila* Cystoblast Differentiation Factor, *benign gonial cell neoplasm*, Is Related to DExH-box Proteins and Interacts Genetically With *bag-of-marbles*. Genetics. 155:1809–1819. doi:10.1093/genetics/155.4.1809. 10.1093/genetics/155.4.1809 PMC1461197 10924476

[jkab312-B33] Ohlstein B , McKearin D. 1997. Ectopic expression of the *Drosophila* Bam protein eliminates oogenic germline stem cells. Development. 124:3651–3662. 9342057 10.1242/dev.124.18.3651

[jkab312-B34] Ote M , Ueyama M, Yamamoto D. 2016. *Wolbachia* protein TomO targets *nanos* mRNA and restores germ stem cells in *Drosophila* *sex-lethal* mutants. Curr Biol. 26:2223–2232. doi:10.1016/j.cub.2016.06.054. 27498563 10.1016/j.cub.2016.06.054

[jkab312-B35] Ote M , Yamamoto D. 2020. Impact of *Wolbachia* infection on *Drosophila* female germline stem cells. Curr Opin Insect Sci. 37:8–15. doi:10.1016/j.cois.2019.10.001. 31726321 10.1016/j.cois.2019.10.001

[jkab312-B36] Le Page DP , Metcalf JA, Bordenstein SR, On J, Perlmutter JI, et al 2017. Prophage WO genes recapitulate and enhance *Wolbachia*-induced cytoplasmic incompatibility. Nature. 543:243–247. doi:10.1038/nature21391. 28241146 10.1038/nature21391 PMC5358093

[jkab312-B37] Rice DW , Sheehan KB, Newton ILG. 2017. Large-scale identification of *Wolbachia pipientis* effectors. Genome Biol Evol. 9:1925–1937. doi:10.1093/gbe/evx139. 28854601 10.1093/gbe/evx139 PMC5544941

[jkab312-B38] Richardson MF , Weinert LA, Welch JJ, Linheiro RS, Magwire MM, et al 2012. Population genomics of the Wolbachia endosymbiont in *Drosophila melanogaster*. PLoS Genet. 8:e1003129.doi:10.1371/journal.pgen.1003129. 23284297 10.1371/journal.pgen.1003129 PMC3527207

[jkab312-B39] Riegler M , Sidhu M, Miller WJ, O'Neill SL. 2005. Evidence for a global Wolbachia replacement in *Drosophila melanogaster*. Curr Biol. 15:1428–1433. doi:10.1016/j.cub.2005.06.069. 16085497 10.1016/j.cub.2005.06.069

[jkab312-B40] Russell SL , Ruelas CJ, Russell SL, Castillo JR. 2020. Chapter 5 trends in symbiont-induced host cellular differentiation. Results Probl Cell Differ. 69:137–176. doi:10.1007/978-3-030-51849-3_5. 33263871 10.1007/978-3-030-51849-3_5 PMC8025664

[jkab312-B41] Schneider DI , Klasson L, Lind AE, Miller WJ. 2014. More than fishing in the dark: PCR of a dispersed sequence produces simple but ultrasensitive *Wolbachia* detection. BMC Microbiol. 14:121.doi:10.1186/1471-2180 24885505 10.1186/1471-2180-14-121 PMC4029913

[jkab312-B2923762] Serga S, , Maistrenko O, , Rozhok A, , Mousseau T, , Kozeretska I. 2014. Fecundity as one of possible factors contributing to the dominance of the *w*Mel genotype of *Wolbachia* in natural populations of *Drosophila melanogaster*. Symbiosis. 63:11–17. doi:10.1007/s13199-014-0283-1.

[jkab312-B42] Serga SV , Maistrenko OM, Matiytsiv NP, Vaiserman AM, Kozeretska IA. 2021. Effects of *Wolbachia* infection on fitness-related traits in *Drosophila melanogaster*. Symbiosis. 83:163–172. doi:10.1007/s13199-020-00743-3.

[jkab312-B43] Shropshire JD , On J, Layton EM, Zhou H, Bordenstein SR. 2018. One prophage WO gene rescues cytoplasmic incompatibility in *Drosophila melanogaster*. Proc Natl Acad Sci USA. 115:4987–4991. doi:10.1073/pnas.1800650115. 29686091 10.1073/pnas.1800650115 PMC5948995

[jkab312-B44] Shropshire JD , Rosenberg R, Bordenstein SR. 2021. The impacts of cytoplasmic incompatibility factor (*cifA* and *cifB* genetic variation on phenotypes. Genetics. 217:1–8. doi:10.1093/genetics/iyaa007. 10.1093/genetics/iyaa007 PMC8218869 33683351

[jkab312-B45] Starr DJ , Cline TW. 2002. A host-parasite interaction rescues *Drosophila* oogenesis defects. Nature. 418:76–79. doi:10.1038/nature00843. 12097909 10.1038/nature00843

[jkab312-B46] Thurmond J , Goodman JL, Strelets VB, Attrill H, Gramates LS, Marygold SJ, et al; FlyBase Consortium. 2019. FlyBase 2.0: the next generation. Nucleic Acids Res. 47:D759–D765. doi:10.1093/nar/gky1003. 30364959 10.1093/nar/gky1003 PMC6323960

[jkab312-B47] Ting X. 2013. Control of germline stem cell self-renewal and differentiation in the *Drosophila* ovary: concerted actions of niche signals and intrinsic factors. Wiley Interdiscip Rev Dev Biol. 2:261–273. doi:10.1002/wdev.60. 24009036 10.1002/wdev.60

[jkab312-B48] Truitt AM , Kapun M, Kaur R, Miller WJ. 2019. *Wolbachia* modifies thermal preference in *Drosophila melanogaster*. Environ Microbiol. 21:3259–3268. doi:10.1111/1462-2920.14347. 10.1111/1462-2920.14347 PMC6766989 29971900

[jkab312-B49] Utarini A , Indriani C, Ahmad RA, Tantowijoyo W, Arguni E, et al; AWED Study Group. 2021. Efficacy of *Wolbachia*-infected mosquito deployments for the control of Dengue. N Engl J Med. 384:2177–2186. doi:10.1056/NEJMoa2030243. 34107180 10.1056/NEJMoa2030243 PMC8103655

[jkab312-B50] Werren JH , Baldo L, Clark ME. 2008. *Wolbachia*: master manipulators of invertebrate biology. Nat Rev Microbiol. 6:741–751. doi:10.1038/nrmicro1969. 18794912 10.1038/nrmicro1969

[jkab312-B51] Zhukova MV , Kiseleva E. 2012. The virulent *Wolbachia* strain wMelPop increases the frequency of apoptosis in the female germline cells of *Drosophila melanogaster*. BMC Microbiol. 12(Suppl. 1):S15. doi:10.1186/1471-2180-12-S1-S15. 22375935 10.1186/1471-2180-12-S1-S15 PMC3287512

