# Supplementary material for: Diverse wMel variants of Wolbachia pipientis differentially rescue fertility and cytological defects of the bag of marbles partial loss of function mutation in Drosophila melanogaster
Source: G3 (Bethesda). 2021 Sep 7;11(12):jkab312. doi: 10.1093/g3journal/jkab312 (PMC8664471; doi:10.1093/g3journal/jkab312)
Supplement: jkab312_Supplementary_Data [file jkab312_supplementary_data.zip › GENETICS-G3-2021-402724-s04.docx]

**Fig S1. Total eggs per female counted over 3 days for the *w^1118^*; *bam*^+^/*bam*^+^ genotype and the *w^1118^*; *bam^L255F^*/*bam^null^* genotype infected with representative *W. pipientis* variants from the *w*Mel-like and *w*MelCS-like clades.** (A) Swarm and Cumming estimation plots showing the total eggs per female for *w^1118^*; *bam*^+^/*bam*^+^ lines infected with *w*Mel59 or *w*MelCSa66. Infection with either variant does not significantly affect total eggs per female. (B) Swarm and Cumming estimation plots showing the total eggs per female for *w^1118^*; *bam^L255F^*/*bam^null^* females infected with the same two *W. pipientis* variants. Infection with either *w*Mel_59 or *w*MelCSa_66 significantly rescues the number of eggs laid per female compared to the uninfected control (95% confidence interval, effect size). Consistent with the results of the nurse cell assay, *w*MelCSa_66 shows a higher rescue effect than *w*Mel_59.
